# Métodos de Inteligência Artificial para Detecção de Fibrilação Atrial Assintomática. Uma Oportunidade para Novas Abordagens de Prevenção e o Papel do Olho do Médico

**DOI:** 10.36660/abc.20240662

**Published:** 2024-11-08

**Authors:** Francisco Darrieux, Tan Chen Wu

**Affiliations:** 1 Hospital das Clínicas da Faculdade de Medicina da Universidade de São Paulo Instituto do Coração São Paulo SP Brasil Instituto do Coração do Hospital das Clínicas da Faculdade de Medicina da Universidade de São Paulo, São Paulo, SP – Brasil

**Keywords:** Inteligência Artificial, Fibrilação Atrial, Programa de Triagem, Análise de Risco de Acidente Vascular Cerebral

A fibrilação atrial (FA) continua sendo a arritmia cardíaca mais comum, associada a um risco cinco vezes maior de acidente vascular cerebral, bem como a um risco maior de insuficiência cardíaca. O tratamento da FA abrange triagem e diagnóstico, melhora da qualidade de vida por meio do controle da frequência ou ritmo, redução da morbidade e mortalidade por meio da prevenção de acidente vascular cerebral e tromboembolismo sistêmico, bem como tratamento de condições associadas. A detecção precoce pode mitigar complicações por meio de intervenção imediata. Infelizmente, a FA geralmente não é reconhecida e tratada porque é frequentemente assintomática ou minimamente sintomática e frequentemente paroxística.

As diretrizes do CCS/CHRS e da ESC recomendam o rastreamento oportunista para FA em pessoas com idade ≥ 65 anos no momento de um encontro médico não relacionado. A justificativa para essas recomendações é baseada em uma combinação de eficácia (a taxa de detecção de FA para pessoas ≥ 65 anos foi relatada como 1,44% (intervalo de confiança [IC] de 95%, 1,13%-1,82%] vs. 0,41% [IC de 95%, 0,31%-0,53%] em pessoas < 65 anos de idade) e os resultados acionáveis (ou seja, pacientes ≥ 65 anos de idade com FA têm indicação de terapia de anticoagulação oral [ACO] para prevenção de AVC).^1^ Além disso, as diretrizes da ESC recomendam triagem sistemática para pacientes com maior risco de AVC (escores CHA2DS2-VA ≥ 2), amplamente baseadas no aumento de 4 vezes na detecção de FA no estudo *Systematic ECG Screening for Atrial Fibrillation Among 75- Year-Old Subjects in the Region of Stockholm and Halland,* Suécia (STROKESTOP), no qual nova FA foi detectada em 3,0% (IC de 95%, 2,7%-3,5%) dos pacientes que participaram de um programa de triagem de 2 semanas em comparação com um eletrocardiograma (ECG) de índice sozinho.^2^

Embora a estratégia de triagem mais comum seja a palpação oportunista do pulso, *smartwatches* habilitados por algoritmos de inteligência artificial (IA) surgiram como ferramentas promissoras para a detecção precoce de FA devido ao seu uso generalizado, facilidade de uso e potencial custo-efetividade. Estudos pragmáticos em larga escala que foram conduzidos para grandes empresas de *smartwatches* como Apple e Fitbit avaliaram a capacidade de triagem de FA na população em geral com base na fotopletismografia. O Apple Heart Study avaliou mais de 400.000 indivíduos (idade média de 41 ± 13 anos, 42% mulheres) para notificação de pulso irregular (NPI), com o Apple Watch indicando possível FA mais um patch de ECG de derivação única para diagnosticar FA.^3^ No geral, apenas 0,52% da população do estudo recebeu uma NPI usando o algoritmo da Apple, com a porcentagem correlacionada com o aumento da idade (≥65 anos: NPI 3,2%). Da mesma forma, o *Fitbit Heart Study* avaliou o desempenho do Fitbit Watch entre mais de 400.000 participantes (idade média de 47 anos, intervalo interquartil de 35 a 58 anos, 71% mulheres), dos quais 1% recebeu uma NPI (≥65 anos: NPI 3,6%).^4^ Daqueles que receberam uma NPI nos estudos Fitbit e Apple Heart, apenas até 25% de todos os participantes notificados nesses estudos retornaram o patch de ECG, e 32,2% e 34,0% dos casos, respectivamente, foram confirmados como tendo FA com duração de pelo menos 30 s no patch de ECG de referência. O valor preditivo positivo (VPP) para FA, confirmado simultaneamente no patch de ECG, do algoritmo da Apple foi de 84,0% (IC de 95%, 76,0%–92,0%). O VPP foi menor entre aqueles com mais de 65 anos, ou seja, 78% (IC de 95%, 64,0%–92,0%). O algoritmo Fitbit produziu uma sensibilidade de 67,6%, especificidade de 98,4% e PPV de 98,2% (IC de 95%, 95,5%–99,5%), com uma ligeira redução entre aqueles com idade ≥65 anos em 97,0% (IC de 95%, 91,4%–99,4%). Entre outros estudos conduzidos em ambientes de pesquisa e entre populações de alto risco para FA, o desempenho dos sensores PPG variou com sensibilidade variando de 87,8% (42) a 94,2% (22) e especificidade de até 99,1%.

Identificar FA entre pacientes assintomáticos de alto risco pode levar ao início de ACO, potencialmente reduzindo o risco de AVC. O estudo eBRAVE-AF mostrou que a triagem baseada em PPG mais que dobrou a taxa de detecção de FA assintomática, levando ao subsequente início de ACO.^5^ Uma metanálise recente demonstrou uma redução significativa no risco de AVC após o início de ACO em pacientes com FA assintomática detectada por dispositivos cardíacos implantáveis.^6^

Outros estudos examinaram o desempenho do ECG inteligente (iECG), um ECG de derivação única baseado em smartwatch com uma função de detecção automática de FA. Mannhart et al. avaliaram a precisão de 5 dispositivos inteligentes na identificação de FA em comparação com um ECG de 12 derivações interpretado por um médico como padrão de referência em uma coorte de pacientes do mundo real. Eles encontraram diferenças no número de traçados inconclusivos e concluíram que, em um ambiente clínico, uma revisão manual dos traçados é necessária em cerca de um quarto dos casos.^7^

Estudos recentes com um modelo de redes de aprendizado profundo (DNN) identificaram pacientes com alto risco de FA de início recente. Attia et al. demonstraram a capacidade de uma DNN de identificar a assinatura eletrocardiográfica de FA paroxística (PxAF) a partir de ECGs de 12 derivações mostrando ritmo sinusal em uma janela de tempo curta.^8^ Raghunath et al. usaram traçados de ECG digitais de 12 derivações de 430.000 pacientes para treinar DNN para prever FA de início recente (dentro de 1 ano). Em pacientes sem histórico de FA que tiveram um AVC relacionado à FA, quase dois terços teriam sido previstos como de alto risco para FA antes do AVC pelo modelo de aprendizado profundo.^9^

Nos últimos anos, uma tecnologia de telemedicina chamada *Stroke Risk Analysis* (SRA) tem analisado um ECG contínuo de 1 hora para identificar pacientes com alto risco de PxAF, mesmo que a análise do ECG não inclua manifestações de FA. O algoritmo foi desenvolvido com base em centenas de conjuntos de dados de pacientes com PxAF e indivíduos sem histórico de FA. Revisões sistemáticas concluem que os resultados obtidos ainda são insuficientes para a tomada de decisão clínica quanto à validade da SRA para prevenção secundária de AVC, mas não excluem a possibilidade de que a técnica tenha potencial para detectar FA e, consequentemente, prevenir novos eventos isquêmicos.^10,11^ Gomes et al., em uma revisão sistemática, mostraram que há evidências de que a SRA tem um valor preditivo negativo significativo (entre 96 e 99,1%) para a detecção de PxAF, apesar da qualidade dos estudos ser classificada como moderada de acordo com o GRADE. Assim, a SRA pode ser uma ferramenta de pré-seleção com implicações para identificar pacientes com maior risco de PxAF como possível causa de AVC que podem se beneficiar do monitoramento cardíaco implantável.^12^

Nesta questão, Andrade et al. avaliaram 120 pacientes ≥ 65 anos com hipertensão ou insuficiência cardíaca com maior risco de FA por SRA que continuaram usando iECG (Kardia) por 7 dias para detectar FA. Em um total de 408 pacientes, 13,7% apresentaram episódios de FA (3,2% por SRA e 10,5% por Kardia). Eles concluíram que a estratégia adotada no estudo RITMO foi eficaz e pode ser útil na identificação de episódios de FA em pacientes assintomáticos com fatores de risco para FA, como hipertensão e insuficiência cardíaca.^13^ No entanto, a ausência de um grupo controle e o diagnóstico baseado apenas em IA, não confirmado pelos clínicos, pode ser preocupante como um farol de uso único na prática clínica. Considerando que o uso do AliveCor Kardia não permite o registro contínuo do ECG, mas sim sob demanda (o que foi bem desenhado no estudo), certamente haverá a possibilidade de não detecção de FA em períodos fora do registro. Outra questão importante no estudo é que ele só foi capaz de detectar FA nos 7 dias de monitoramento "não contínuo", não excluindo a possibilidade de ocorrência de FA em períodos posteriores a uma semana.

O diagnóstico se torna mais desafiador no contexto de episódios assintomáticos, ou FA detectada em dispositivos de monitoramento de longo prazo, particularmente aqueles que não fornecem um ECG. Para se proteger contra o diagnóstico inadequado de FA, a diretriz ESC de 2024 publicada recentemente para FA continua a recomendar que a documentação de ECG por ECG padrão de 12 derivações ou dispositivos de derivação única e múltipla, confirmada por médicos, seja necessária para iniciar a estratificação de risco e o gerenciamento de FA.^14^

## References

[1] Cheung CC, Nattel S, Macle L, Andrade JG (2021). Management of Atrial Fibrillation in 2021: An Updated Comparison of the Current CCS/CHRS, ESC, and AHA/ACC/HRS Guidelines. Can J Cardiol.

[2] Svennberg E, Friberg L, Frykman V, Al-Khalili F, Engdahl J, Rosenqvist M (2021). Clinical Outcomes in Systematic Screening for Atrial Fibrillation (STROKESTOP): a Multicentre, Parallel Group, Unmasked, Randomised Controlled Trial. Lancet.

[3] Lubitz SA, Faranesh AZ, Selvaggi C, Atlas SJ, McManus DD, Singer DE (2022). Detection of Atrial Fibrillation in a Large Population Using Wearable Devices: The Fitbit Heart Study. Circulation.

[4] Avram R, Ramsis M, Cristal AD, Nathan V, Zhu L, Kim J (2021). Validation of an Algorithm for Continuous Monitoring of Atrial Fibrillation Using a Consumer Smartwatch. Heart Rhythm.

[5] Rizas KD, Freyer L, Sappler N, von Stülpnagel L, Spielbichler P, Krasniqi A (2022). Smartphone-based Screening for Atrial Fibrillation: A Pragmatic Randomized Clinical Trial. Nat Med.

[6] McIntyre WF, Benz AP, Becher N, Healey JS, Granger CB, Rivard L (2024). Direct Oral Anticoagulants for Stroke Prevention in Patients with Device-Detected Atrial Fibrillation: A Study-Level Meta-analysis of the NOAH-AFNET 6 and ARTESiA Trials. Circulation.

[7] Mannhart D, Lischer M, Knecht S, Lavallaz JF, Strebel I, Serban T (2023). Clinical Validation of 5 Direct-to-consumer Wearable Smart Devices to Detect Atrial Fibrillation: BASEL Wearable Study. JACC Clin Electrophysiol.

[8] Attia ZI, Noseworthy PA, Lopez-Jimenez F, Asirvatham SJ, Deshmukh AJ, Gersh BJ (2019). An Artificial Intelligence-enabled ECG Algorithm for the Identification of Patients with Atrial Fibrillation During Sinus Rhythm: A Retrospective Analysis of Outcome Prediction. Lancet.

[9] Raghunath S, Pfeifer JM, Ulloa-Cerna AE, Nemani A, Carbonati T, Jing L (2021). Deep Neural Networks Can Predict New-onset Atrial Fibrillation from the 12-Lead ECG and Help Identify Those at Risk of Atrial Fibrillation-Related Stroke. Circulation.

[10] Kishore A, Vail A, Majid A, Dawson J, Lees KR, Tyrrell PJ (2014). Detection of Atrial Fibrillation after Ischemic Stroke or Transient Ischemic Attack: A Systematic Review and Meta-analysis. Stroke.

[11] Korompoki E, Del Giudice A, Hillmann S, Malzahn U, Gladstone DJ, Heuschmann P (2017). Cardiac Monitoring for Detection of Atrial Fibrillation after TIA: A Systematic Review and Meta-analysis. Int J Stroke.

[12] Gomes RAF, Sá MPBO, Montenegro MV, Furtado LCC, Costa JHCFD, Coutinho DB (2024). Is Stroke Risk Analysis (SRA) a Reliable Method for Predicting Atrial Fibrillation? A Systematic Review. PLoS One.

[13] Andrade RP, Vitorino PVO, Sousa ALL, Miranda RD, Nogueira BAA, Cestário EES (2024). A Program to Optimize the Detection of Paroxysmal Atrial Fibrillation: The RITMO Study. Arq Bras Cardiol.

[14] van Gelder IC, Rienstra M, Bunting KV, Casado-Arroyo R, Caso V, Crijns HJGM (2024). 2024 ESC Guidelines for the Management of Atrial Fibrillation Developed in Collaboration with the European Association for Cardio-thoracic Surgery (EACTS). Eur Heart J.

