# Supplementary material for: Métodos de Inteligência Artificial para Detecção de Fibrilação Atrial Assintomática. Uma Oportunidade para Novas Abordagens de Prevenção e o Papel do Olho do Médico
Source: Arq Bras Cardiol. 2024 Nov 8;121(9):e20240662. [Article in Portuguese] doi: 10.36660/abc.20240662 (PMC11634225; doi:10.36660/abc.20240662)
Supplement: Translation

# Translation: Artificial Intelligence Methods for Detecting Asymptomatic Atrial Fibrillation. An Opportunity for New Prevention Approaches and the Role of the Doctor's Eye

**DOI:** 10.36660/abc.20240662i

**Authors:** Francisco Darrieux, Tan Chen Wu

**Affiliations:** 1 São Paulo SP Brazil Instituto do Coração do Hospital das Clínicas da Faculdade de Medicina da Universidade de São Paulo, São Paulo, SP – Brazil

**Keywords:** Artificial Intelligence, Atrial Fibrillation, Screening Program, Sroke Risk Analysis

Atrial fibrillation (AF) remains the most common cardiac arrhythmia, associated with a fivefold increased risk of stroke, as well as an increased risk for heart failure. The management of AF encompasses screening and diagnosis, improvement of quality of life through rate or rhythm control, reduction of morbidity and mortality through prevention of stroke and systemic thromboembolism, as well as treatment of associated conditions. Early detection may mitigate complications through prompt intervention. Unfortunately, AF is often unrecognized and untreated because it is frequently asymptomatic or minimally symptomatic and often paroxysmal.

Both the CCS/CHRS and ESC guidelines advocate opportunistic screening for AF in people ≥ 65 years of age at the time of an unrelated medical encounter. The rationale for these recommendations is based on a combination of effectiveness (the AF detection rate for persons ≥ 65 years has been reported as 1.44% (95% confidence interval [CI], 1.13%-1.82%] vs 0.41% [95% CI, 0.31%-0.53%] in persons < 65 years of age), and the actionable outcomes (ie, patients ≥ 65 years of age with AF indicate oral anticoagulation [OAC] therapy for stroke prevention)^1^. In addition, the ESC guidelines recommend systematic screening for patients with higher risk of stroke (CHA2DS2-VA scores ≥ 2), largely based on the 4-fold increase in detection of AF in the Systematic ECG Screening for Atrial Fibrillation Among 75- Year-Old Subjects in the Region of Stockholm and Halland, Sweden (STROKESTOP) study, in which new AF was detected in 3.0% (95% CI, 2.7%-3.5%) of patients participating in a 2-week screening program compared with an index electrocardiogram (ECG) alone.^2^

Although the most common screening strategy is opportunistic pulse palpation, smartwatches empowered by artificial intelligence (AI) algorithms have emerged as promising tools for early detection of AF due to their widespread use, easiness of use, and potential cost-effectiveness. Large-scale, pragmatic studies that were conducted for major smartwatch companies like Apple and Fitbit assessed the ability to screen for AF in the general population relying on photoplethysmography. The Apple Heart Study evaluated over 400,000 individuals (mean age 41 ± 13 years, 42% women) for irregular pulse notification (IPN), with the Apple Watch indicating possible AF plus a single-lead ECG patch to diagnose AF.^3^ Overall, only 0.52% of the study population received an IPN using the Apple algorithm, with the percentage correlating with increased age (≥65 years: IPN 3.2%). Similarly, the Fitbit Heart Study assessed the performance of the Fitbit Watch among over 400,000 participants (median age 47 years, interquartile range 35–58 years, 71% women), of whom 1% received an IPN (≥65 years: IPN 3.6%).^4^ Of those who received an IPN in the Fitbit and Apple Heart studies, only up to 25% of all notified participants in these studies returned the ECG patch, and 32.2% and 34.0% of the cases, respectively, were confirmed to have AF lasting at least 30 s on the reference ECG patch. The positive predictive value (PPV) for AF, confirmed concurrently on the ECG patch, of the Apple algorithm was 84.0% (95% CI, 76.0%–92.0%). The PPV was lower among those over 65, i.e., 78% (95% CI, 64.0%–92.0%). The Fitbit algorithm yielded a sensitivity of 67.6%, specificity of 98.4%, and PPV of 98.2% (95% CI, 95.5%–99.5%), with a slight reduction among those aged ≥65 years at 97.0% (95% CI, 91.4%–99.4%). Among other studies conducted in research settings and among populations at high risk for AF, the performance of PPG sensors varied with sensitivity ranging from 87.8% (42) to 94.2% (22) and specificity up to 99.1%.

Identifying AF among high-risk, asymptomatic patients could lead to the initiation of OAC, potentially reducing the risk of stroke. The eBRAVE-AF trial showed that PPG-based screening more than doubled the detection rate of asymptomatic AF, leading to subsequent OAC initiation.^5^ A recent meta-analysis demonstrated a significant reduction in stroke risk after OAC initiation in patients with asymptomatic AF detected by implantable cardiac devices.^6^

Other studies examined the performance of intelligent ECG (iECG), a smartwatch-based single-lead ECG with an automatic AF detection function. Mannhart et al. assess the accuracy of 5 smart devices in identifying AF compared with a physician-interpreted 12-lead ECG as the reference standard in a real-world cohort of patients. They found differences in the number of inconclusive tracings and concluded that in a clinical setting, a manual review of tracings is required in about one-fourth of cases.^7^

Recent studies with a deep learning networks (DNN) model identified patients at high risk for new-onset AF. Attia et al. demonstrated the ability of a DNN to identify the electrocardiographic signature of paroxysmal AF (PxAF) from 12-lead ECGs showing sinus rhythm in a short time window.^8^ Raghunath et al. used 12-lead digital ECG traces from 430000 patients to train DNN to predict new-onset AF (within 1 year). In patients with no history of AF who have an AF-related stroke, nearly two-thirds would have been predicted to be high-risk for AF before the stroke by the deep learning model.^9^

In recent years, a telemedicine technology called Stroke Risk Analysis (SRA) has analyzed a 1-hour continuous ECG to identify patients at high risk of PxAF, even if the ECG analysis does not include manifestations of AF. The algorithm was developed based on hundreds of datasets of patients with PxAF and individuals with no history of AF. Systematic reviews conclude that the results achieved are still insufficient for clinical decision-making regarding the validity of SRA for secondary stroke prevention but do not exclude the possibility that the technique has the potential to detect AF and, consequently, prevent new ischemic events.^10,11^ Gomes et al., in a systematic review, showed that there is evidence that the SRA has a significant negative predictive value (between 96 and 99.1%) for the detection of PxAF despite the quality of the studies being classified as moderate according to GRADE. Thus, SRA can be a pre-selection tool with implications for identifying patients at higher risk of PxAF as a possible cause of stroke who may benefit from implantable cardiac monitoring.^12^

In this issue, Andrade et al. evaluated 120 patients ≥ 65 years with hypertension or heart failure at higher risk of AF by SRA who continued using iECG (Kardia) for 7 days to detect AF. In a total of 408 patients, 13.7% presented episodes of AF (3.2% by SRA and 10.5% by Kardia). They concluded that the strategy adopted in the RITMO study was effective and may be useful in identifying episodes of AF in asymptomatic patients with risk factors for AF, like hypertension and heart failure.^13^ However, the lack of a control group and the diagnosis based only on AI, not confirmed by clinicians may be of concern as a single-use beacon in clinical practice. Considering that the use of AliveCor Kardia does not allow continuous recording of the ECG but rather on demand (which was well designed in the study), there will certainly be the possibility of non-detection of AF in periods outside of recording. Another important issue in the study is that it was only able to detect AF in the 7 days of "non-continuous" monitoring, not excluding the possibility of AF occurring in periods subsequent to one week.

Diagnosis becomes more challenging in the context of asymptomatic episodes, or AF detected on longer-term monitoring devices, particularly those that do not provide an ECG. To guard against the inappropriate diagnosis of AF, the recently published 2024 ESC guideline for AF continues to recommend that ECG documentation by standard 12-lead ECG or single- and multiple-lead devices, confirmed by physicians, is required to initiate risk stratification and AF management.^14^
